# Synthesis, surface activity, and corrosion inhibition capabilities of new non-ionic gemini surfactants

**DOI:** 10.1038/s41598-024-57853-x

**Published:** 2024-04-05

**Authors:** M. A. Deyab, Ibrahim Z. Ibrahim, Omnia A. A. El-Shamy, Khalil A. Khalil, Abdelhamid F. Awad, Majed M. Alghamdi, Adel A. El-Zahhar, Mohamed A. Abo-Riya

**Affiliations:** 1 Egyptian Petroleum Research Institute (EPRI), Nasr City, Cairo Egypt; 2 Chemistry Department, Faculty of Science, Zagazig University, Zagazig, Egypt; 3 Chemistry Department, Faculty of Science, Benha University, Benha, 13518 Egypt; 4 Department of Chemistry, College of Science, King Khalid University, P.O. Box 9004, 61413 Abha, Saudi Arabia

**Keywords:** Gemini surfactants, Surface activity, Corrosion, Theoretical studies, Chemistry, Physical chemistry

## Abstract

Several environmentally acceptable non-ionic gemini surfactants are synthesized in this work using natural sources, including polyethenoxy di-dodecanoate (GSC12), polyethenoxy di-hexadecanoate (GSC16), and polyethenoxy di-octadecenoate (GSC18). The produced surfactants are confirmed by spectrum studies using FT-IR, ^1^HNMR, and ^13^CNMR. It explored and examined how the length of the hydrocarbon chain affected essential properties like foaming and emulsifying abilities. Surface tension examinations are used to assess the surface activity of the examined gemini surfactants. The lower value of critical micelle concentrations (0.381 × 10^−4^M) is detected for GSC18. Their spontaneous character is shown by the negative values of the free energy of adsorption (ΔG_ads_) and micellization (ΔG_mic_) which arranged in the order GSC18 > GSC16 > GSC12. Based on theoretical, weight loss, and electrochemical investigations, these novel surfactants were investigated for their possible use in inhibiting carbon steel from corroding in 1 M HCl. Measuring results show that GSC18 inhibits corrosion in carbon steel by 95.4%. The isotherm of adsorption evaluated for the investigated inhibitors and their behavior obeys Langmuir isotherm.

## Introduction

Surfactants are unique molecules that contain both lipophilic and lipophobic moieties^1–3^. These compounds have great importance in many industrial applications, such as detergents, floatation, additives, paints, paste control, and corrosion inhibitors^3–6^. Modern surfactants, known as gemini surfactants, comprise two typical surfactant molecules chemically bonded together using a flexible or stiff spacer^7,8^. Critical micelle concentrations (CMCs) of gemini surfactants are generally 10–100 times lower than those of equivalent monomeric surfactants^9,10^. Dimeric surfactants outperform their monomeric counterparts in terms of their ability to reduce surface tension. Gemini surfactants' peculiar characteristics are the basis for their use as emulsifiers, dispersants, coating agents, and corrosion inhibitors^11^.

Metal corrosion leads to many problems in the industry and exposes factories to significant losses and environmental risks such as pollution^12–14^. One of the essential substances used in the construction of pipelines for gas and oil transportation is carbon steel^15,16^. The primary alloying element of these iron-based materials is carbon, which makes steel alloy hard, so there are differences in strength and hardness according to the percentage of carbon in the alloy^17^. Hydrochloric acid (HCl) is widely used in several sectors, including the acidifying process of petroleum tanks for storage and chemical cleaning, pickling, and the purpose of descale procedures^18^.

Utilizing corrosion inhibitors on metal surfaces is a widespread method for reducing metal corrosion. Nitrogen, Sulphur, and oxygen atoms should be present in molecules that act as acid inhibitors^19^. The inhibitor molecules protect the metal from the corrosive environment by forming an adsorption coating on the metal's surface. For an inhibitor to be efficient, water needs to be extracted from the metal surface area before it can engage with the anodic and cathodic reactions^20–22^.

Gemini surfactants inhibit corrosion by adsorbing the polar group (hydrophilic head) on the metal surface, where the surfactant's non-polar group (hydrophobic tail) is directed toward the solution. The effect of the alkyl chain length of different pyridyl gemini surfactants was studied. The authors concluded that a long hydrophobic chain enhances the corrosion performance of the P110 steel in acid media^23^. The presence of a surfactant inhibitor drastically alters the properties of surfaces and interfaces because surfactants may combine to produce micelles^24^.

Deyab et al. examined newly synthesized gemini surfactants based on alkyl benzenaminium with different alkyl chain lengths^25^. The authors concluded that excellent inhibition efficiency ranged from 95 to 99%. In addition, the effect of spacer length for a series of cationic gemini surfactants was studied by El-Shamy and Nissem^26^. The results declare that the inhibition efficiency increases linearly with spacer length.

The usage of eco-friendly corrosion inhibitors is a concern^27^. Abdallah et al. investigate the inhibition efficiencies of different natural extracts^28^. The results demonstrate that the applied natural extracts acted as pitting corrosion inhibitors and the potential has shifted to great noble values.

Numerous studies claim that the inhibitor's functional groups, steric effects, electronic density of the donor atoms, the orbital character of the donating electrons, and other physical, chemical, and electronic characteristics mainly determine the inhibitory action^29^. Quantum chemical calculations are a potential method that supports understanding of the molecule structure, electrical structure, and reactivity of the corrosion inhibitors^30^. DFT has introduced a beneficial framework based on theoretical calculations. Quantum chemical parameters are calculated by Al-Fahemi et al. for different investigated inhibitors. The results proven that their action are excellent as corrosion inhibitors^31^.

The work involved the preparation of new, cost-effective, and eco-friendly nonionic gemini surfactants based on natural sources, the synthesized gemini surfactants characterized by FT-IR, ^1^HNMR, and ^**1**3^CNMR spectroscopy techniques. The surface activities, foam test, and emulsification performance for the synthesized geminis are determined and discussed. The protection efficiency of these surfactants for carbon steel in 1 M HCl is investigated using electrochemical and weight loss measurements. In addition, the effect of electronic properties on the corrosion resistance of the synthesized surfactants is evaluated using quantum calculations by applying density function theory (DFT).

## Experimental part

### Materials

Dodecanoic, hexadecanoic, octadecanoic acid, di bromo ethane, polyethylene glycol (400), and benzene were obtained from Sigma Aldrich, and p. toluenesulfonic acid, ethyl alcohol, potassium hydroxide purchased from Al-nasr chemical company. 1.0 M hydrochloric acid was used as an aggressive corrosion medium (blank solution). In this study, the carbon-steel specimens for each test were prepared using a different range of emery sheets with sizes ranging from 400 to 2500. X-ray fluorescence (Bruker) was used to determine the composition of the working electrode. The composition (weight %) of carbon steel includes 0.36% carbon, 0.04% phosphorus, 0.09% silicon, 0.48% manganese, and the remaining element is iron.

### Synthesis

#### Preparation of monoester

The monoesters of different hydrophobic chain lengths were prepared according to the following steps. 0.1 mol of the fatty acid (dodecenoic acid, hexadecenoic acid, and octadec-9-enoic acid) was dissolved separately in toluene and mixed with equimolar polyethylene glycol (400)^32^.

Dean Stark's apparatus was used, outfitted with a magnetic stirrer to facilitate the reaction. The mixture was heated until the eliminated water content in Dean Stark was 1.8 mL. The polyethylene glycol mono laurate and palmitate are colorless, viscous liquids with 94% and 93% yields (weight %). A pale yellow liquid with a high viscosity and a yield of 92% (weight %) was obtained for the synthesized mono oleate. The formed molecules of polyethylene glycol monolaurate, monopalmitate, and monooleate were characterized using FTIR and ^1^HNMR analyses. The deuterium solvent used in NMR analysis is dimethyl sulfoxide.

#### Synthesis of gemini surfactants

**Figure 1:**
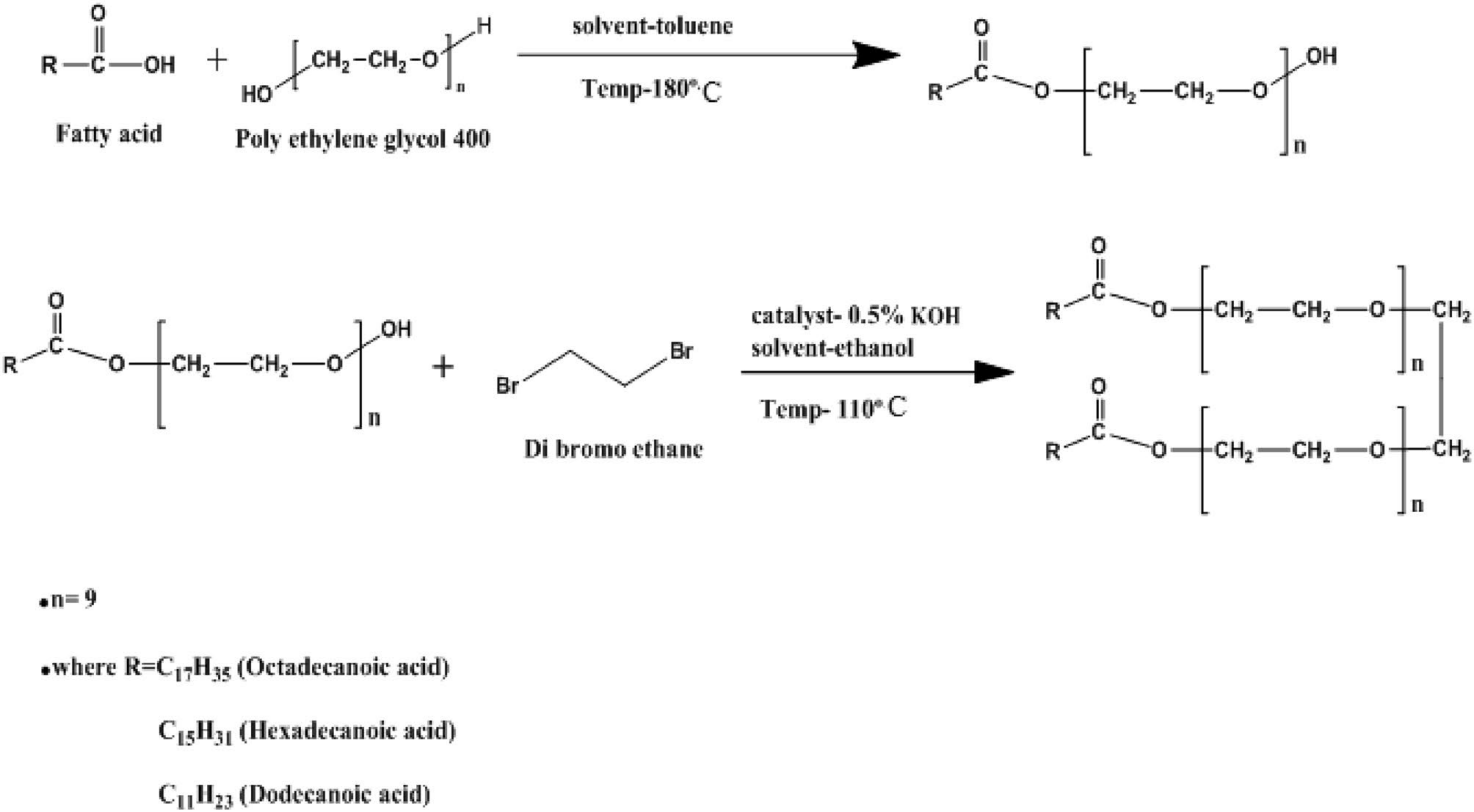
Synthesis of ethoxylated monoester nonionic surfactants and nonionic gemini surfactants.

The synthesized mono-esters prepared geminis (GSC12, GSC16, and GSC18) by adding dibromo ethane and stirring in ethanolic KOH for 36 h at 110 °C. Then, the solvent was evaporated, and the mixture was repeatedly washed with diethyl ether to remove the unreacted material. Geminis surfactants were recrystallized using petroleum ethers to yield of 95%. IR and ^1^HNMR spectra proved the prepared compounds. Figure 1 shows the synthesis of the investigated gemini surfactants.

### Surfactants properties measurements

#### Emulsification power and stability

The surfactant solution's emulsifying ability at ambient temperature was evaluated using liquid paraffin, palm, castor, and pine oils, according to the following^31^: 20 mL of the surfactant solution (0.1% w/v) was poured into the 100 mL cylinder. Then, paraffin, palm oil, castor oil, and pine oil, totalling 20 mL, were transferred to the measuring cylinder. The cylinder was inverted five times at a rate of once per minute. Subsequently, the cylinders were set upright. It was determined how long it would take to filter 19 mL of aqueous solution.

#### Foam’s strength and steadiness

The modified Ross-Miles method was used for determining the foaming power of a 0.1% aqueous surfactant solution by measuring the height of the foam five minutes after a vigorous 100 shakes at 298 K. By comparing the foam height after 5 min to the initial value, foam stabilities were calculated^33^.

By using the modified Ross-Miles method, the foaming power of a 0.1% aqueous solution of surfactant was determined by measuring the height of the foam five minutes after shaking vigorously 100 times at a temperature of 298 K. To calculate the foaming stability, the foam height after 5 min was compared to the initial foam height.

#### Surface tension

The surface tension of the geminis surfactant solution was determined using a Du-nouy Tensiometer. The device was calibrated with de-ionized water before measurements and found around 72 at 298 K^34^. Different concentrations of the synthesized gemini surfactants range from 1 × 10^−6^ to 8 × 10^−3^ M.

### Electrochemical and chemical measurements

A glass unit (100 mL with three electrodes) was utilized for the electrochemical analysis. A platinum (Pt) plate was employed as the counter electrode, while a saturated calomel electrode (SCE) was the reference electrode. The carbon steel working electrode has a contact area of 0.266 cm^2^. The Gamry-reference 3000 potentiostat/galvanostat was used for all experiments. Potential current graphs were performed under specific conditions (scan rate = 0.125 mV s^−1^, potential region =  ± 250 mV vs. open circuit potential (OCP). The polarization experiments were conducted according to ASTM G59-97(2020). The electrochemical impedance spectroscopy (EIS) was conducted at A 10 mV peak-to-peak sinusoidal wave in the 100 kHz–0.01 Hz frequency region. Impedance measurements in the lower frequency range are often used to analyze the behavior of corrosion processes that involve slow electrochemical reactions, such as the formation and dissolution of protective films, or the diffusion of ions through passive layers. In the higher frequency range, impedance measurements are useful for studying more rapid electrochemical processes, including charge transfer reactions at the metal-electrolyte interface and double-layer capacitance effects.

Evaluation of weight loss was carried out according to ASTM G 01. The following relation determines the corrosion rate of carbon steel (C_R_):

1 $${\text{C}}_{{\text{R}}} = {\text{W/A}} \times {\text{t}}$$

where W is mass loss (mg), A is the surface area of specimens (cm^2^) and t is the immersion time (h).

Before each experiment, the carbon steel surface was manually scraped away with increasing grit silicon carbide (SiC) sheets, then ultrasonically cleaned in ethanol, thoroughly rinsed with water, and air dried.

The surface morphology investigations were conducted using ZEISS/EVO Scanning Electron Microscope (SEM) for carbon steel samples after 24 h of immersion in 1 M HCl solution in the absence and presence of 10 × 10^−3^ M surfactants at 298 K.

### Quantum studies

The quantum chemical calculations were performed using the HyperChem 8.010 program implemented in the core i7 laptop. Density functional Theory (DFT) was selected to evaluate the electronic properties of the investigated surfactants after complete geometry optimization. B3LYP/medium 6-31G basis set.

## Results and discussion

### Confirmation of surfactants structures

**Figure 2:**
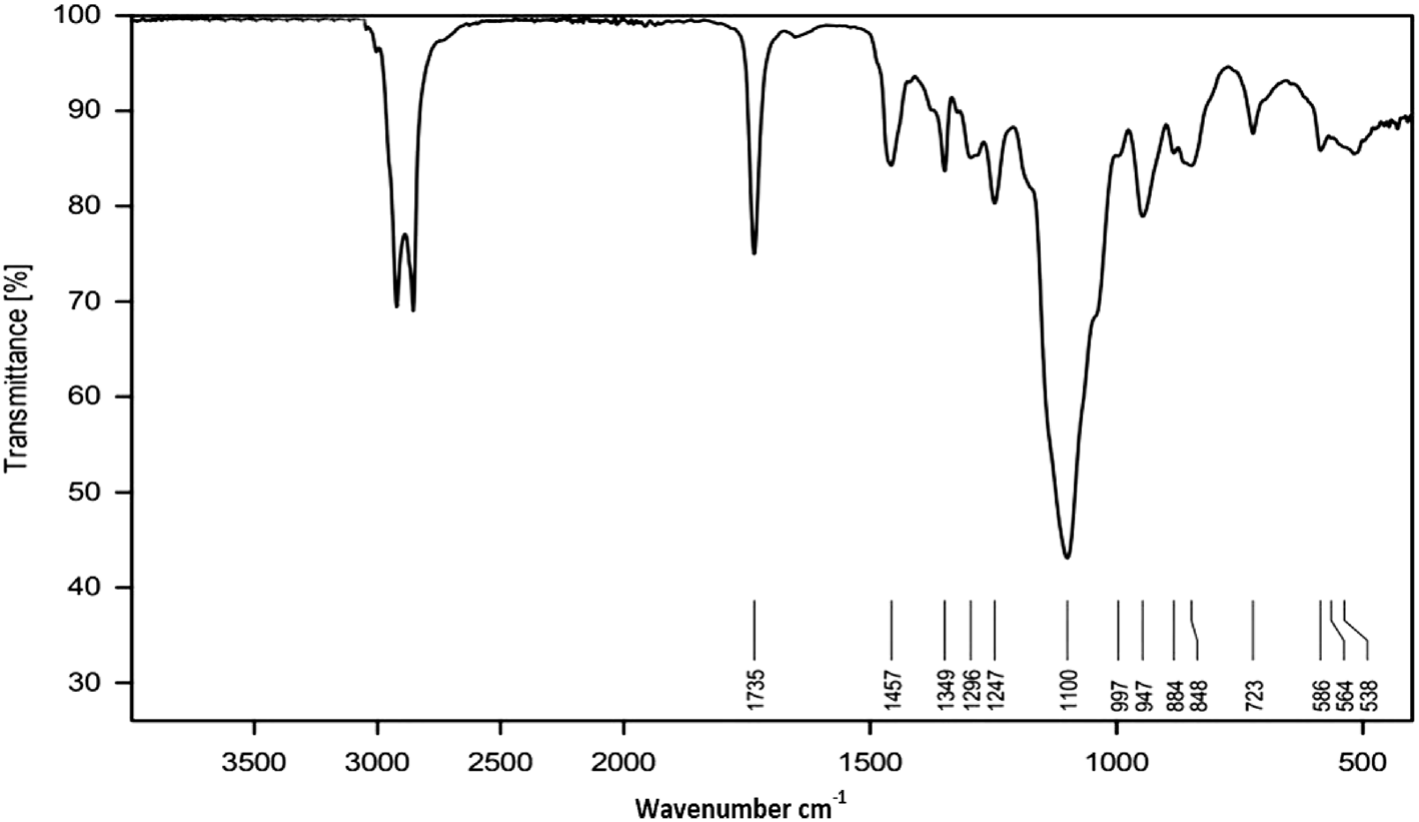
FTIR spectra of the synthesized GSC12.

Figure 2 shows FT-IR spectra of GSC12. The absorption bands at 1735 cm^−1^ and 1100 cm^−1^, related to the stretching vibration of C=O and C–O, respectively, confirm the monolaurate molecule's formation. In addition, the bands at 2925 and 2859 cm^−1^ are assigned to the stretching vibration of C-H of the hydrophobic tail of the synthesized surfactants, while their stretching bending is located at 1457 cm^−1^ and 1349 cm^−1^. Similar characteristic bands (See Fig. S1a,b confirm the formation of GSC16 and GSC18.

**Figure 3:**
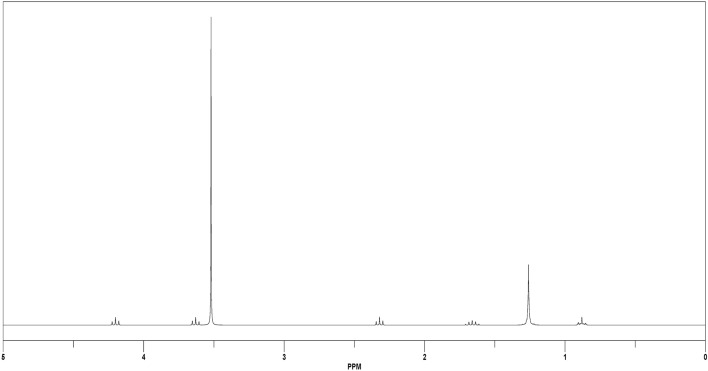
^1^HNMR spectra of GSC12.

Figure 3 shows ^1^H NMR spectra of the synthesized GSC12. Concerning the figure, it is clear that characteristic spectra appear as the following: δ (ppm): 0.88 (t, 3H, [**CH**_**3**_–CH_2_–]), 1.26 (S, 16H, **[(CH**_**2**_**)**_**8**_**–**]), 1.66 (m, 2H, [**CH**_**2**_CH_2_COO]), 2.32 (t, 2H, [**CH**_**2**_–COO]), 3.52 (s, 32H, 8[**CH**_**2**_**–CH**_**2**_–O]), 3.63 (t, 2H,[COO–CH_2_–**CH**_**2**_–O–]), 4.20(t, 2H, [COO–**CH**_**2**_–CH_2_–O–]). Also, the similar bands in Fig. S1a,b are related to GSC16 and GSC18.

**Figure 4:**
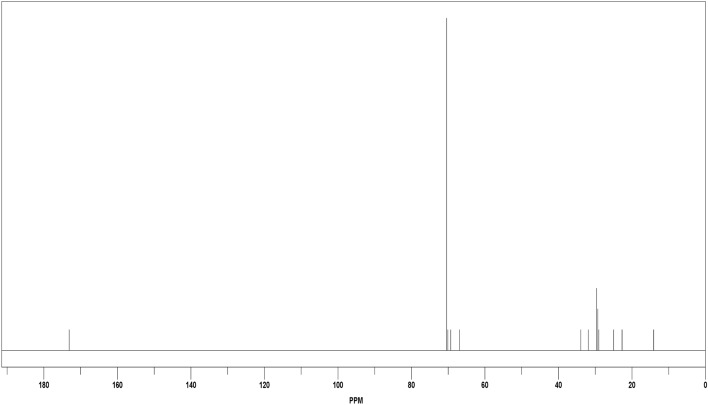
^13^CNMR spectra of GSC12.

Figure 4 shows the ^13^C NMR spectra of GSC12, the signal (δ) (present at 14.1 ppm) corresponds to the terminal CH_3_ group. The aliphatic CH_2_ of the long-chain hydrocarbon is located at δ of 22.70, 29.25, and 33.90 ppm. In addition, the signals at 69.9 and 70.1 ppm are assigned to the CH_2_ group, which is linked to the carboxylate group. The signal corresponding to the carboxyl group's carbon is detected at δ = 173.1 ppm. The similar signals are detected for GSC16 and GSC18 (Fig. S3).

In the case of GSC18, additional signals located at 27.7 and 130.6 ppm correspond to CH_2_ (nearest ethylene group) and CH of the ethylene group, respectively. The FT-IR, ^1^HNMR, and ^13^CNMR matched the chemical structure of the synthesized gemini surfactants (GSC12, GSC16, and GSC18).

### Surface activities

#### Emulsification power and emulsification stability:

Detergents, petrochemicals, and cosmetics are a few significant industrial applications where emulsifying power is utilized. The chemical nature of the oil and the surfactant has an essential effect on the stability of the generated emulsion. With the help of liquid paraffin, palm, castor, and pine oil in water, the long-term stability of the emulsion between the surfactant solution and the oil can be assessed.

**Figure 5:**
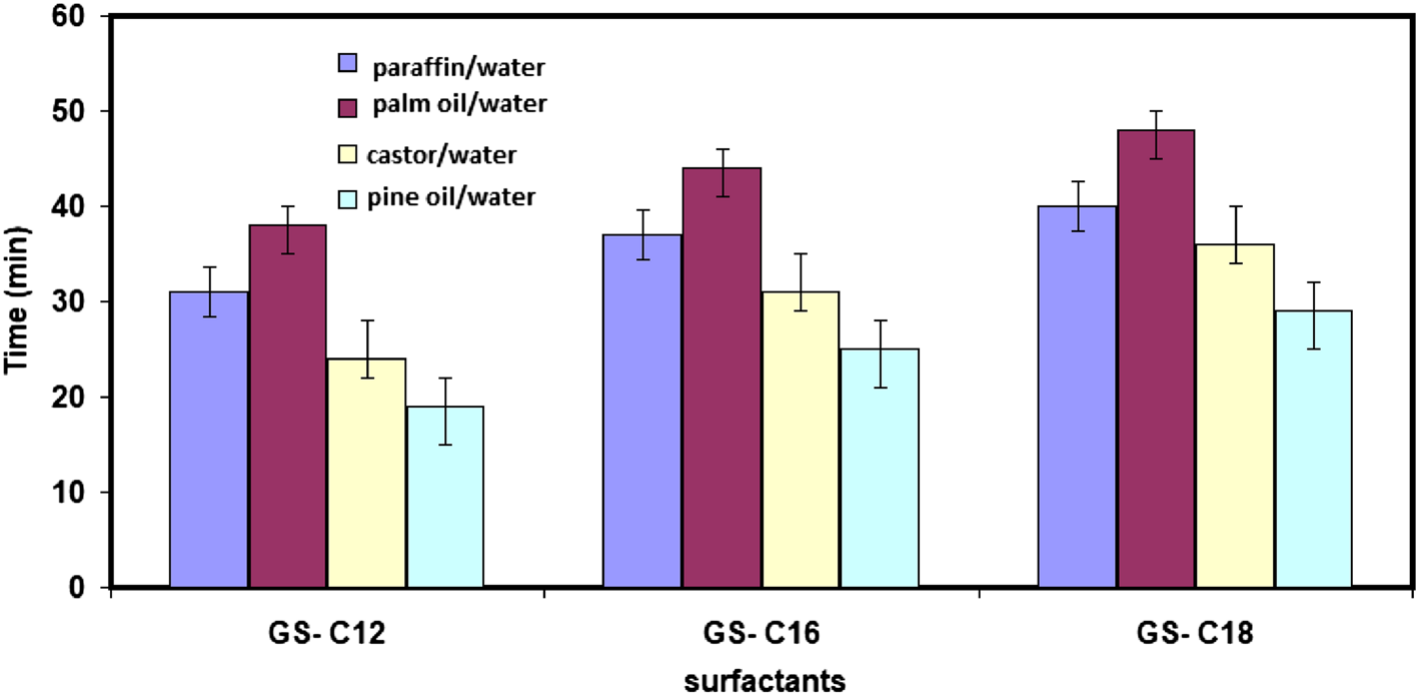
Emulsifying ability of an aqueous solution of synthetic non-ionic gemini surfactants at a concentration of 0.1% (w/v) for a variety of oils.

The produced surfactant has good emulsification stability, particularly peg oleate, which demonstrated the highest degree of emulsification power due to its high affinity for adsorption at the interface, as shown by their values for C_cmc_ (critical micelle concentration) and adsorption-free energy. The emulsification power and emulsification stability values are shown in Fig. 5. The figure shows the emulsifying ability of an aqueous solution of synthetic non-ionic gemini surfactants at a concentration of 0.1% (w/v) for a variety of oils.

#### Foam's strength and steadiness

The power and stability of the foam results are listed in Table 1. It was noted that all the prepared gemini compounds initially have good foam, but the foam level decreases as time increases. Longer hydrocarbon chains result in less foaming action, and the foaming stability becomes good, so gemini laurate has maximum foaming ability and lowest strength.

**Table 1:** The stability and foaming performance of a synthetic non-ionic gemini surfactant solution at 0.1% (w/v) in water.

Gemini surfactants	Foam height (mL)		Foam stability 100%
	Initial (v_1_)	5 min (v_2_)	
GSC12	42	30	71.4%
GSC16	39	29	74.3%
GSC18	35	27	77.7%

#### Surface tension and critical micelle concentration (CMC)

**Figure 6:**
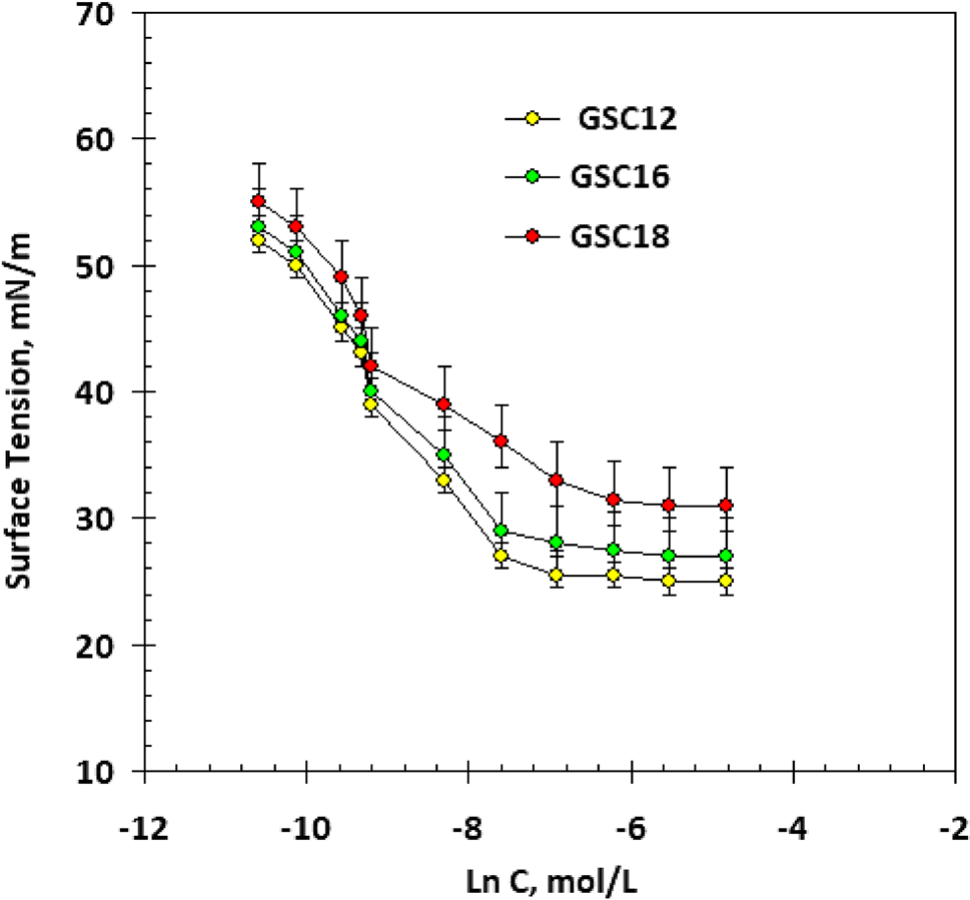
Relation between surface tension and ln C of the synthetic non-ionic gemini surfactants at 298 K.

The surface tension of the gemini solutions was less than that of pure bi-distilled water. Surfactant molecules adsorb at the water–air interface, causing the hydrophobic tails to point towards the air phase while the polar head groups remain attached to the water surface. There is less surface tension because fewer hydrogen bonds are formed between water molecules in the presence of adsorbed surfactant molecules at the air-solution interface. Synthetic surfactants can be evaluated for their surface-active features by measuring their surface tension at varying concentrations. Figure 6 displays the logarithmic surfactant concentrations versus surface tension relationship at 298 K. The CMC was calculated. GSC18 revealed the lowest CMC values because more methylene groups are in the hydrophobic chains, which results in more repulsion between surfactant molecules. So, as the hydrophobic chain length increases, there is a greater tendency for the molecules to form micelles in the bulk of the solution^35^.

- Effectiveness π_cmc_
  When surface tension was reduced between bi-distilled water and prepared nonionic gemini surfactants at critical micelle concentration, it was defined as effectiveness and expressed by equation^36^:
  2 $$\pi_{{{\text{cmc}}}} = \gamma_{{\text{o}}} - \gamma_{{{\text{cmc}}}}$$
  here, *γ*_*o*_ is the surface tension of the bi-distilled water (71.8 mN/m), and *γ*_*cmc*_ is the surface tension of the surfactant solution at the CMC. The calculated effectiveness values for the geminis are listed in Table 2. GSC18 caused more significant surface reduction at CMC than GSC12 and GSC16 due to its highest hydrophobic characteristics^37^.

**Table 2:** Gemini nonionic surfactants surface and thermodynamic characteristics.

Surfactant	CMC × 10^−3^ (M)	*γ*_*c*_ (mN m^−1^)	*π*_*cmc*_ (mN m^−1^)	*Γ*_*max*_ (mol cm^−2^)	***A***_min_ (nm^2^)	ΔG_mic_ (kJ mol^−1^)	ΔG_ads_ (kJ mol^−1^)
GSC12	0.0912 ± 0.0004	28	44	4.5	0.369	− 22.56	− 23.57
GSC16	0.0746 ± 0.0004	31	41	4.7	0.353	− 23.05	− 23.95
GSC18	0.0381 ± 0.0003	34	38	4.8	0.345	− 24.68	− 25.50

#### The Excess of the surface (Γ_max_)

Surface excess (Γ_max_) was determined based on the surfactant adsorption at the air/water interface. The Gibbs adsorption equation was used to calculate the Γ_max_^37^.

3 $$\Gamma_{\max } = ( - 1/RT)({\text{d}}\gamma /{\text{d}}\ln {\text{C}})$$

where *R* is the universal gas constant, *T* is absolute temperature, (dγ/d lnC) is the slope of the linear line on the surface tension graph. As the hydrophobicity increases from C12 to C18, efficient coverage of the interface surface results in a higher value of surface excess concentration (see Table 2). The previous results agree with other research^37,38^.

#### The surface area per molecule (A_min_)

Adsorbed molecules require the smallest possible surface area, *A*_min_, the amount of space one molecule occupies at the liquid/air interface in units of nm^2^. It was determined using the following equation^38^.

4 $$A_{min} = {1}0^{{{14}}} {/}N_{{\text{A}}} \Gamma_{max}$$

where NA is the Avogadro’s number, from the results (see Table 2), it was clear that (A_min_) values decrease and *Γ*_*max*_ increase by increasing the hydrophobic chain, explained by the tendency of the coiling of the long hydrophobic tails as mentioned before in different research^39,40^.

Since the hydrophobic part hates polar water solvents, the surfactants' tendency to form micelles increases as the hydrophobic chain length increases, and the micelles will start at lower concentrations (see Table 2). In addition, the remarkable ability of the long hydrophobic for coiling decreases their minimum surface area (A_min_) and hence increases the surface excess (*Γ*_*max*_) concentration and, therefore, the surface tension (*π*_*cmc*_)^41,42^. In sum, the synthesized geminins possess excellent surface activities, facilitating their adsorption to the metal surface.

#### Thermo-dynamic properties of the synthesized gemini non-ionic surfactants

Surfactant molecules tended to adsorb at the interface or micellize in the bulk of their solution. The free energy of micellization (ΔG_mic_) was calculated by calculating CMC values from the micellization Gibbs equation:

5 $$\Delta G_{{{\text{mic}}}} = {2}.{3}0{3}RT\left( {{1} - {\text{n}}} \right){\text{logCMC}}$$

where n is the number of counter ions in the case of ionic surfactant n = 0 for nonionic surfactants.

Free energy of adsorption ΔG_ads_ were calculated by the following equation:

6 $$\Delta {\text{G}}_{{{\text{ads}}}} = \Delta {\text{G}}_{{{\text{mic}}}} - 0.{623} \times \pi_{{{\text{cmc}}}} A_{{{\text{min}}}}$$

where ΔG_mic_ is micellization-free energy in KJ mole^−1^n, π_cmc_ is effectiveness in mN/m and **(**A_min_). The smallest area of surface per adsorbed molecule in nm^2^^38^. Since the values in micellization-free energy and surfactant adsorption are always negative, the process happened spontaneously. G_ads_ have a lower value than G_mic_, which is a more negative value. Thus, the produced surfactant was more likely to adsorb at the air/water interface than to form micelles in most of its solutions. Adsorption and micellization-free energy increase negatively by increasing hydrophobic chain length for surfactants^36^.

### Anti-corrosion characteristics of gemini surfactants

#### Electrochemical and weight loss studies

**Figure 7:**
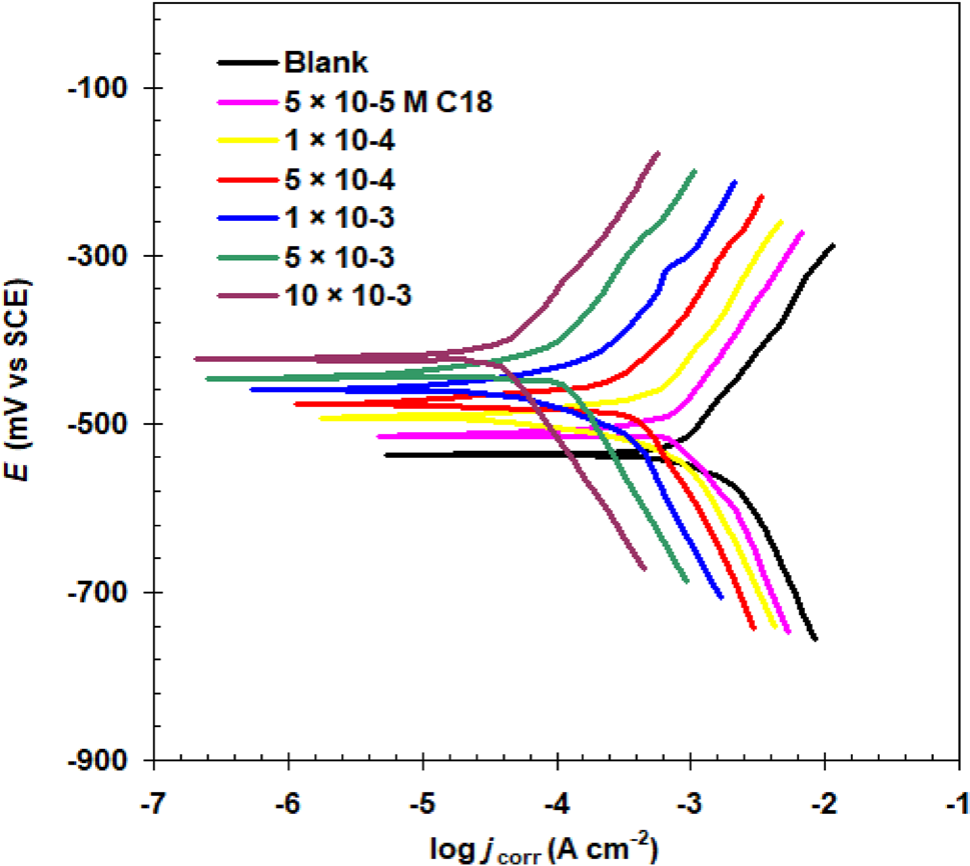
Graphical representations of carbon steel's potentiodynamic polarisation in 1.0 M HCl at 298 K with and without different concentrations of GSC18.

The kinetic behaviors of steel corrosion reactions in 1.0 M HCl solution with GSC12, GSC16, and GSC18 gemini surfactants were investigated using electrochemical study results. The polarization graph for the GSC18 is shown, for example, in Fig. 7. The plot is shaped like a Tafel. Table 3 shows the potential for corrosion (*E*_corr_), Tafel slopes (*β*_a_ and *β*_c_), and corrosion current density (*j*_corr_) as polarization parameters. The following relationship is utilized to calculate the efficiency of protection (*P*_j_%)^43,44^.

7 $$P_{{\text{j}}} \% = \frac{{j_{{{\text{corr}}(0)}} - j_{{{\text{corr}}}} }}{{j_{{{\text{corr}}(0)}} }} \times 100$$

**Table 3:** Carbon steel's polarization features and the efficiency of inhibition by new surfactants in a 1.0 M HCl solution.

Cpd	Conc M	*E*_corr_ (mV vs SCE)	*j*_corr_ (μAcm^−2^)	*β*a (mV dec^−1^)	− *β*c (mV dec^−1^)	P_j_%
	Blank	− 537 ± 1.2	784.0 ± 3.5	95.2	124.9	–
GSC12	5 × 10^−5^ 1 × 10^−4^ 5 × 10^−4^ 1 × 10^−3^ 5 × 10^−3^ 10 × 10^−3^	− 530 ± 1.1 − 522 ± 1.3 − 503 ± 0.9 − 498 ± 1.8 − 483 ± 2.0 − 479 ± 1.7	631.9 ± 2.8 575.4 ± 4.4 304.2 ± 3.7 228.1 ± 2.9 119.9 ± 1.9 80.7 ± 2.3	91.4 90.9 88.2 81.9 78.3 80.3	120.4 145.8 132.7 117.3 104.9 111.4	19.4 26.6 61.2 70.9 84.7 89.7
GSC16	5 × 10^−5^ 1 × 10^−4^ 5 × 10^−4^ 1 × 10^−3^ 5 × 10^−3^ 10 × 10^−3^	− 523 ± 1.1 − 510 ± 2.5 − 498 ± 1.9 − 484 ± 1.3 − 480 ± 0.9 − 473 ± 1.7	594.2 ± 5.5 544.0 ± 3.4 275.9 ± 1.8 221.8 ± 2.5 88.6 ± 3.6 62.7 ± 4.6	93.5 87.4 68.3 77.8 71.5 70.9	127.4 120.4 119.4 134.8 99.8 114.6	24.2 30.6 64.8 71.7 88.7 92.0
GSC18	5 × 10^−5^ 1 × 10^−4^ 5 × 10^−4^ 1 × 10^−3^ 5 × 10^−3^ 10 × 10^−3^	− 515 ± 1.8 − 494 ± 2.2 − 476 ± 1.9 − 460 ± 1.9 − 446 ± 1.2 − 424 ± 1.3	575.9 ± 5.1 501.3 ± 3.8 229.4 ± 2.8 173.6 ± 3.7 69.2 ± 1.1 35.4 ± 1.3	87.4 75.8 88.3 92.3 89.6 82.5	137.3 126.3 133.9 145.2 129.6 122.3	26.5 36.0 70.7 77.8 91.1 95.4

The corrosion current density in a blank acid solution is given by *j*_corr(0)_.

Table 3 includes the following details:

1. By incorporating GSC12, GSC16, and GSC18, j_corr_ values are significantly reduced to very low levels^44^.
2. *E*_corr_ changes are insignificant (less than 85 mV) in the control sample. This demonstrates that all of the GSC12, GSC16, and GSC18 are of mixed nature^45–47^.
3. *E*_corr_ started to shift anodically concerning the blank at all gemini surfactant concentrations. This implies that these additives are mixed inhibitors with predominant anodic activity^48,49^.
4. Significant percentage inhibition efficiencies were achieved with low quantities of gemini surfactant (i.e., 10 × 10^−3^ M). This is attributed to their ability to form protective films on metal surfaces, which act as a barrier against corrosive species. The inhibition efficiency has also improved with increasing gemini surfactant concentration.
5. The results show that the inhibition efficiency values for GSC12, GSC16, and GSC18 differ markedly, where the inhibition efficiency of gemini surfactants is shown in the following order: GSC12 < GSC16 < GSC18.
6. At the highest concentration of GSC18 (10 × 10^−3^ M), the optimal performance (95.4%) was observed.

The fundamental cause of the gradual reduction in corrosion of C-steel specimens in 1.0 M HCl is the ability of novel gemini surfactants GSC12, GSC16, and GSC18 to adsorb on steel surface^50–52^. Gemini surfactants work together to block cathodic and anodic reactions.

Given that the C-steel has been positively charged by either an inhibited or uninhibited 1.0 M HCl solution, the gemini surfactant molecules are always able to adsorb on the Fe/solution interface in at least one form^53–55^:

(1) Back–forward interactions occur among the bi-e’s and vacant 3d of the metal surface^56^. (2) Free oxygen pairs of e’s and 3d of carbon steel^57^. (3) interaction between positively charged cloud located over carbonyl groups and 3d.

The inhibition effectiveness improves with the length of the terminal chain (i.e., GSC12 < GSC16 < GSC18). This is clarified by implying that increasing the length of the terminal chain tends to increase the extent of surface coverage and the average area surrounded by each adsorbed molecule.

**Figure 8:**
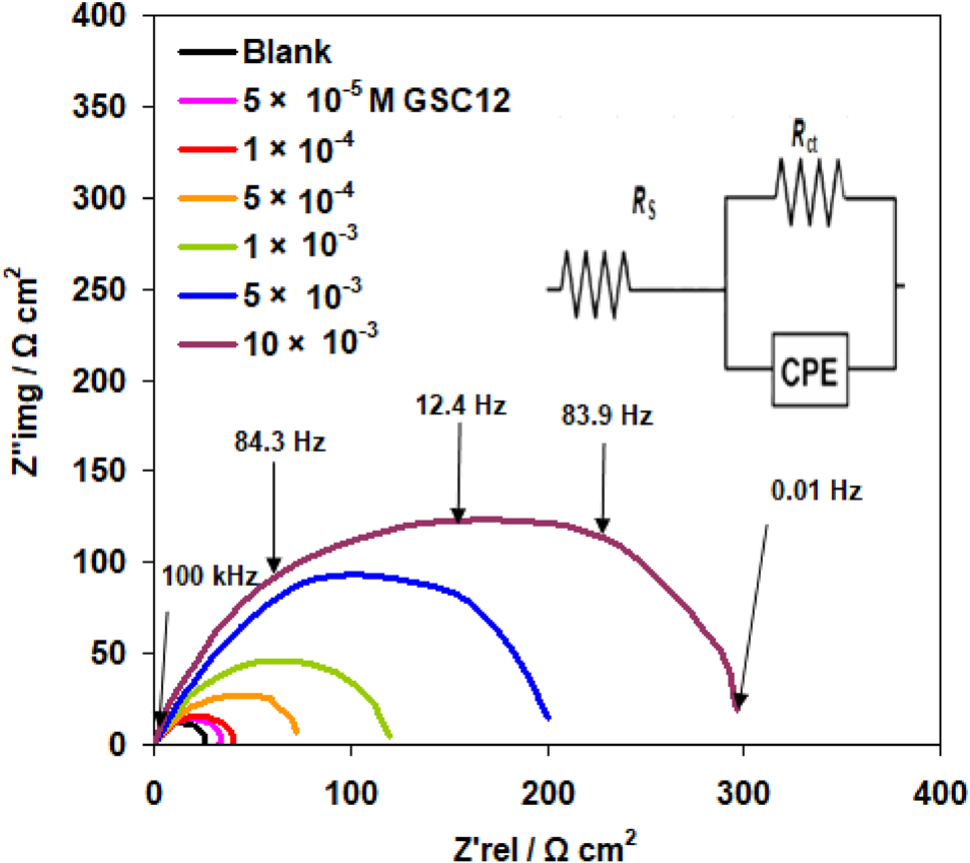
Nyquis plots for carbon steel in 1.0 M HCl at 298 K with and without different concentrations of GSC12.

At 298 K, carbon steel was subjected to electrochemical impedance spectroscopy after exposure to 1.0 M HCl. The Nyquist graphs for increasing amounts of GSC12 (as an example) are shown in Fig. 8. The diameter of semi-circles grows with the addition of newly synthesized surfactants. This is frequently related to a charge transfer mechanism and an improvement in the surface resistivity of carbon steel. The comparable equivalent circuit with a charge transfer resistance (*R*_ct_), a constant phase element (CPE), and solution resistance (*R*_s_) is depicted in Fig. 8 (insert image).

Table 4 shows the numerical values of the several EIS parameters (*R*_ct_ and CPE) as well as the inhibition efficiency (*η*_R_%). The *η*_*R*_% from EIS data is given by^43^:

8 $$\eta_{{\text{R}}} \% = \frac{{R_{{{\text{ct}}}} - R_{{{\text{cto}}}} }}{{R_{{{\text{ct}}}} }} \times 100$$

where *R*_cto_ = charge transfer resistances in absence of new surfactants.

The *R*_ct_ increases significantly when GSC12, GSC16, and GSC18 concentrations increase, but the CPE drops considerably. This significant drop could be caused by a growth in the thickness of the electrical double layer (due to surfactant compounds surface adsorption) and/or a reduction in the local dielectric constant^48^. EIS demonstrates the inhibitory effectiveness of gemini surfactants to follow the same patterns as polarization (see Tables 3 and 4).

**Table 4:** Carbon steel's EIS features and the efficiency of inhibition by new surfactants in 1.0 M HCl solution.

Cpd	Conc M	*R*_*ct*_ (Ω cm^−2^)	*CPE* (µF cm^−2^)	*η*_*R*_ (%)
	Blank	25.61 ± 1.3	75.9	
GSC12	5 × 10^−5^ 1 × 10^−4^ 5 × 10^−4^ 1 × 10^−3^ 5 × 10^−3^ 10 × 10^−3^	33.47 ± 0.9 37.33 ± 1.2 75.54 ± 2.4 121.37 ± 2.8 197.00 ± 2.5 304.88 ± 2.9	64.9 50.3 30.1 20.2 10.9 3.9	23.5 31.4 66.1 78.9 85.7 91.6
GSC16	5 × 10^−5^ 1 × 10^−4^ 5 × 10^−4^ 1 × 10^−3^ 5 × 10^−3^ 10 × 10^−3^	37.06 ± 1.7 40.07 ± 1.5 89.23 ± 2.1 124.92 ± 2.7 264.02 ± 3.2 483.20 ± 4.1	61.0 42.3 25.6 14.2 4.7 1.6	30.9 36.1 71.3 79.5 90.3 94.7
GSC18	5 × 10^−5^ 1 × 10^−4^ 5 × 10^−4^ 1 × 10^−3^ 5 × 10^−3^ 10 × 10^−3^	39.82 ± 1.1 44.61 ± 1.1 121.95 ± 2.0 152.44 ± 2.2 350.82 ± 2.1 985.00 ± 4.2	57.8 40.7 19.3 10.5 1.3 0.56	35.7 42.6 79.0 83.2 92.7 97.4

Table 5 shows the carbon steel corrosion rate (the result obtained from weight loss studies) after 24 h immersion in 1.0 M HCl solution in the absence and presence of 10 × 10^−3^ M surfactants at 298 K and 328 K. The presence of newly synthesized surfactants in a 1.0 M HCl solution decreases the corrosion rate of carbon steel C_R_, demonstrating that they have corrosion inhibiting properties. The corrosion-inhibition effectiveness of surfactants (*ƞ*_w_%) is determined from weight loss data employing a given formula^20^:

9 $$\eta_{{\text{w}}} \% = (C_{{{\text{R}}0}} - C_{{\text{R}}} )/C_{{{\text{R}}0 }} \times {1}00$$

*C*_R0_ = corrosion rate without surfactants.

Table 5 shows that the inhibiting corrosion efficacy of surfactants based on weight loss data follows the same patterns as electrochemical investigations.

**Table 5:** Corrosion rate (weight loss data) and inhibition efficiency values for carbon steel in 1.0 M HCl solution in the absence and presence of 10 × 10^−3^ M of surfactants at 298 K and 328 K.

Temperature (K)	Solution	*C*_R_ (mg cm^−2^ h^−1^)	*ƞ*_w_%
298	Blank	6.33 ± 0.4	–
	10 × 10^−3^ M GSC12	1.12 ± 0.03	82.3
328	Blank	9.23 ± 0.6	–
	10 × 10^−3^ M GSC12	1.91 ± 0.04	79.32
298	Blank	6.33 ± 0.4	–
	10 × 10^−3^ M GSC16	0.64 ± 0.06	89.8
328	Blank	9.23 ± 0.6	–
	10 × 10^−3^ M GSC16	1.52 ± 0.03	83.5
298	Blank	6.33 ± 0.4	–
	10 × 10^−3^ M GSC18	0.32 ± 0.02	94.8
328	Blank	9.23 ± 0.6	–
	10 × 10^−3^ M GSC18	0.81 ± 0.03	91.2

Furthermore, the inhibitory efficacy diminishes slowly as temperature rises (Table 5), indicating a physisorption mechanism^28^.

**Figure 9:**
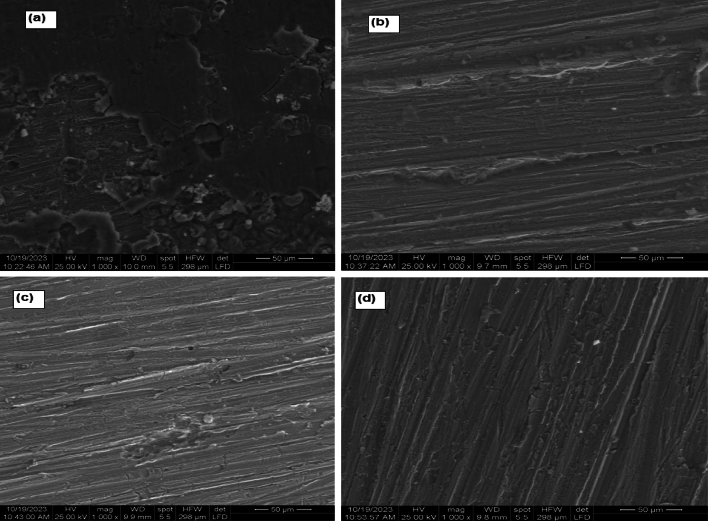
SEM for carbon steel in 1.0 M HCl solution in the absence (micrograph **a**) and presence of GSC12 (micrograph **b**), GSC16 (micrograph **c**) and GSC18 (micrograph **d**) at 298 K.

The morphological inspection (SEM) of the carbon steel in 1.0 M HCl solution in the absence and presence of 10 × 10^−3^ M of surfactants at 298 K are presented in Fig. 9. In the blank solution (micrograph a), the surface morphology of carbon steel exhibited structural damage and intense roughness on top. In the presence of 10 × 10^−3^ M of surfactants (micrograph b, c, and d), the carbon steel has a clean surface and is corrosion-free.

#### Quantum studies

The highest occupied molecule orbital energy, E_HOMO_, and the lowest vacant molecular orbital energy, E_LUMO_, were calculated and then employed in the following equation to derive other essential quantum parameters for the gemini surfactants^31,54,55,58^.

10 $${\text{Energy gap}}\left( {\Delta {\text{E}}_{{\text{g}}} } \right) = {\text{E}}_{{{\text{HOMO}}}} - {\text{E}}_{{{\text{LUMO}}}}$$

11 $${\text{Global hardness }}\left( \eta \right) = - 0.{5}({\text{E}}_{{{\text{HOMO}}}} - {\text{E}}_{{{\text{LUMO}}}} )$$

12 $${\text{Energy of back}} - {\text{donnation }}\left( {{\text{E}}_{{{\text{b}} - {\text{d}}}} } \right) = \eta /{4}$$

**Figure 10:**
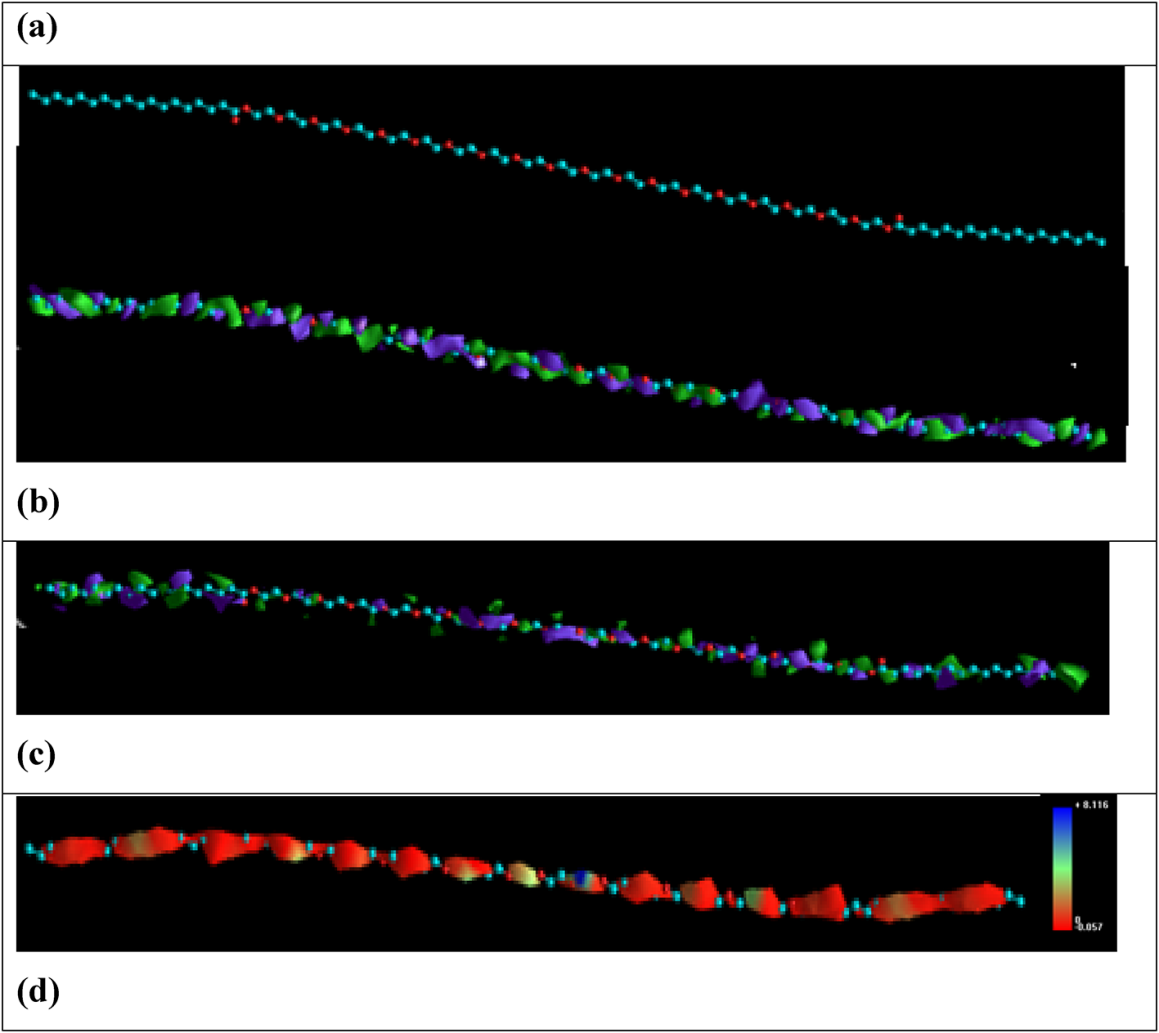
GSC18 (**a**) after optimization, (**b**) HOMO, (**c**) LUMO, and (**d**) Molecular electrostatic potential.

An inhibitor's efficiency might be affected by its electrical and geometric molecular structure. The frontier orbital theory states that the HOMO and LUMO orbitals of the reactants were the primary sites of reaction, and an interaction between their frontier orbitals causes a transition state to arise. To explore the inhibitory mechanism, looking into the distribution of HOMO and LUMO was crucial. Figure 10, Figs. S4, and Fig. S5 show the optimized structure, HOMO, LUMO, and molecular electrostatic potential for the synthesized surfactants.

The molecular structure and ability to donate and receive electrons are determined by the E_HOMO_ and E_LUMO_ values, respectively (see Table 6). In addition, it was reported that lower energy gap (ΔE_g_) values would result in excellent inhibition efficiency since the molecule needs little energy to remove an electron from the final occupied orbital^59^. The values of ΔE_g_ of the investigated gemini surfactants are arranged in the following order: GSC12 ≈ GSC16 > GSC18.

**Table 6:** Quantum chemical parameter for the gemini surfactants.

	E_HOMO_ (eV)	E_LUMO_ (eV)	ΔE_g_ (eV)	η (eV)
GSC12	− 10.563	1.073	11.636	5.818
GSC16	− 10.564	1.072	11.636	5.818
GSC18	− 10.578	1.056	11.634	5.817

GSC18 possesses the lowest ΔE_g_ and highest E_HOMO_ values, indicating strong adsorption ability onto the steel surface via donation and back-donation interaction^60^. In addition, the global hardness (η) of the investigated inhibitors was calculated, and GSC18 possesses the lowest value. It is known that a hard molecule has less tendency to adsorption^61^. In contrast to hard molecules, soft molecules can more easily supply electrons to the metal surface, also due to their low energy gap value ΔE_g_ values. Consequently, the molecule's reactive site may be absorbed, where has the maximum value^62^. Furthermore, all the investigated molecule possesses similar (≈ 1.454 eV) and lower back donation energy, indicating the remarkable ability of these molecules to not only donate electrons to the vacant d orbital of carbon steel but also accept an electron from metals to give a stable, protective layer^59^. The previous electronic parameter recommended all the investigated geminis as corrosion inhibitors and GSC18 is the most efficient.

#### Adsorption studies

Electrochemical studies are carried out to assess the surface coverage (*ɵ*) at various gemini surfactant concentrations (*C*_inh_), and several isotherms are used to identify the best fit that characterizes the inhibitor molecules' behavior. The Langmuir isotherm is being proven to be the best at representing the adsorption process. Monolayer surface coverage is predicted by the Langmuir-adsorption model to follow an asymptotic procedure.

The following relationships reflect this isotherm and the Gibbs free energy change (Δ*G*^0^_ads_)^61^:

13 $$\frac{{C_{{{\text{inh}}}} }}{\theta } = \frac{1}{{K_{{{\text{ads}}}} }} + C_{{{\text{inh}}}}$$

14 $$\Delta G_{{{\text{ads}}}}^{0} = - RT \, ln(55.5K_{ads} )$$

**Figure 11:**
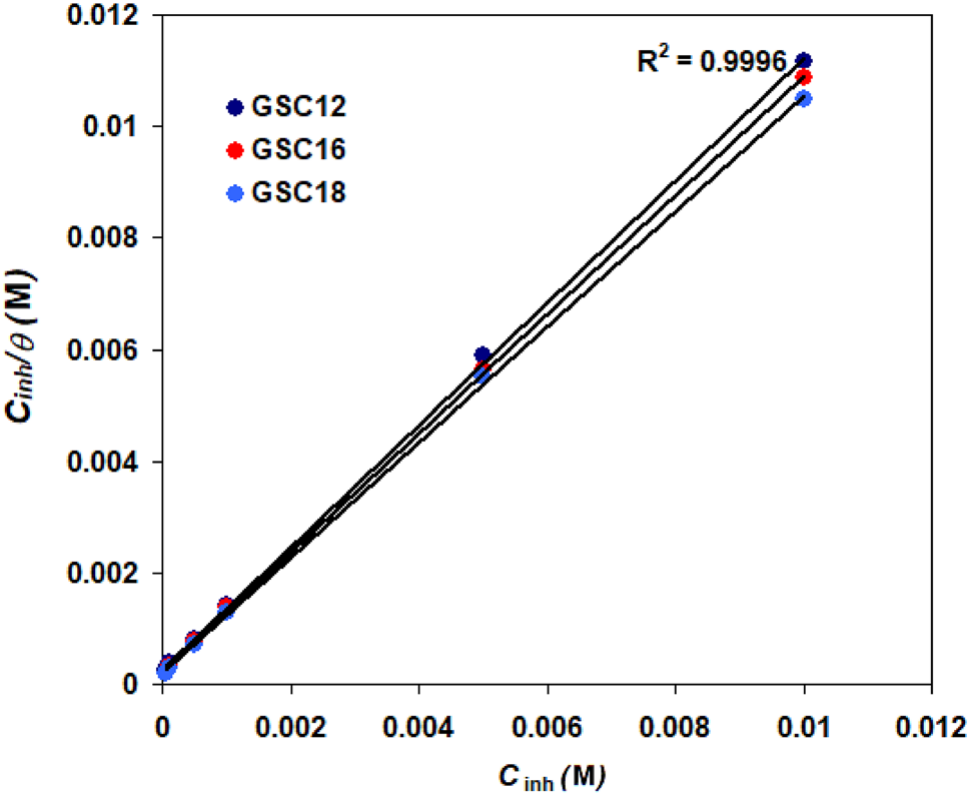
Langmuir isotherm for the studied gemini surfactants at 298 K.

*R* = 8.314 J mol^−1^ K^−1^, *T* = the thermodynamic temperature in Kelvin. *K*_ads_ = adsorption constant. Figure 11 shows the Langmuir adsorption isotherm for the adsorption of GSC12, GSC16, and GSC18 on the carbon steel surface. Linear regression coefficients R^2^ are approximate to one (R^2^ = 0.996). The values of *K*_ads_ are 3.3 × 10^3^, 5 × 10^3^_,_ 5.6 × 10^3^ M^−1^ for GSC12, GSC16, and GSC18, respectively. The value of Δ*G*^0^_ads_ for GSC12, GSC16 and GSC18 were found to be -29.97, -31.0, and -31.28 kJ mol^−1^. Δ*G*^0^_ad_ is negative, indicating that the GSC12, GSC16, and GSC18 molecules have a high inclination to be adsorbed onto the steel surface and that the film produced is steady^62^. The absolute values of Δ*G*^0^_ads_, falling within the range of -20 into − 40 kJ mol^−1^, suggest that GSC12, GSC16, and GSC18 adsorption involves physisorption and chemisorption^62^.

## Conclusion

Three nonionic gemini surfactants with different hydrophobic chain lengths (GSC12, GSC16, and GSC18) were prepared, and their emulsifying and foaming power were investigated. Surface tension measurements are used to determine the surface activities of the synthesized surfactant solutions. The data show that increasing the hydrophobic characters decreases the CMC value and other surface parameters (*π*_*cmc*_, Γ_max_, and A_min_). In addition, from thermodynamic studies, GSC18 has the highest ability for both micellization and adsorption. The corrosion inhibition efficiency was examined using electrochemical and weight loss measurements and the results declare efficient inhibition for all surfactants in the following order: GSC18 > GSC16 > GSC12. Moreover, DFT is applied to relate the electronic properties of the synthesized gemini surfactants by calculating different quantum descriptors as energy gaps using practical data. Both theoretical and experimental studies demonstrate the high efficacy of all gemini surfactants.

### Supplementary Information

Supplementary Figures.

## Data Availability

The datasets used and/or analysed during the current study available from the corresponding author on reasonable request.
