# Supplementary material for: Synthesis, surface activity, and corrosion inhibition capabilities of new non-ionic gemini surfactants
Source: Sci Rep. 2024 Apr 5;14:8040. doi: 10.1038/s41598-024-57853-x (PMC10997777; doi:10.1038/s41598-024-57853-x)
Supplement: Supplementary file 1 — Supplementary Figures. [file 41598_2024_57853_MOESM1_ESM.docx]

Supplementary data


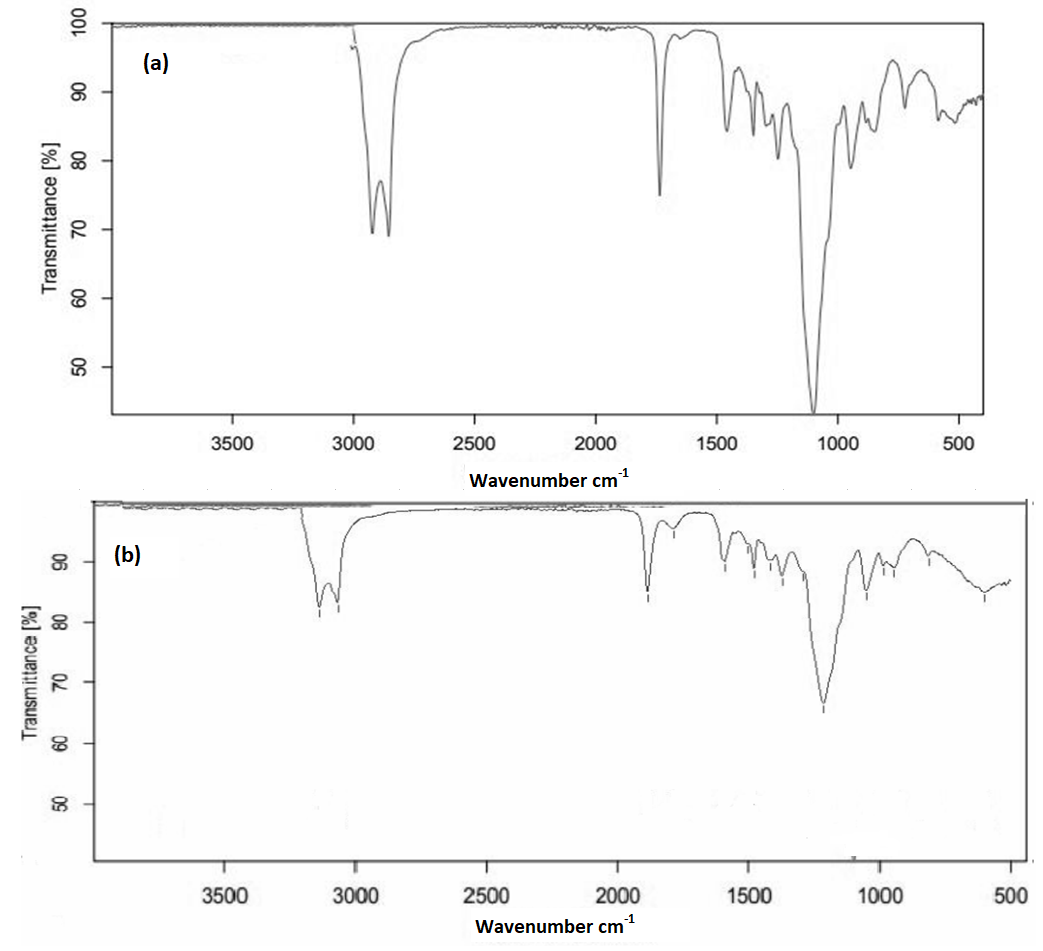


Fig. S1 FTIR spectra of (a) GSC16 and (b) GSC18


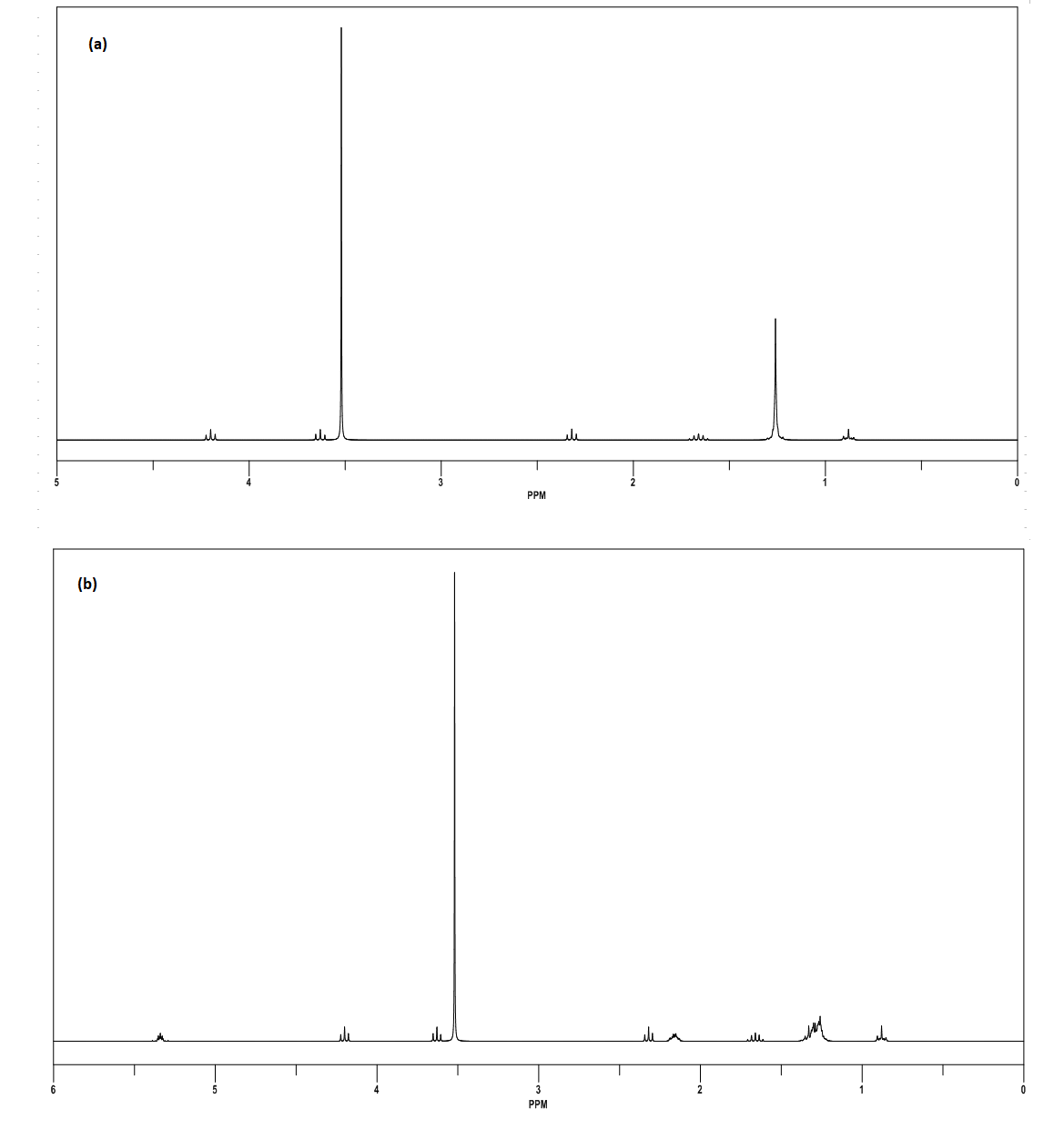


**Fig. S2:** ^1^HNMR spectra of (a) GSC16 and (b) GSC18


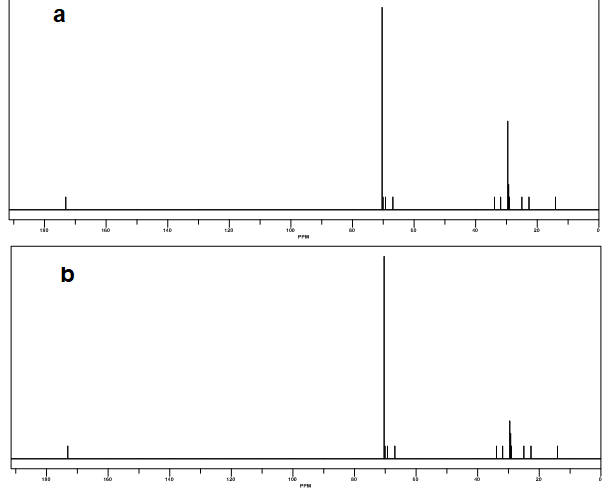


Fig. S3: ^13^CNMR spectra of (a) GSC16 and (b) GSC18.

| 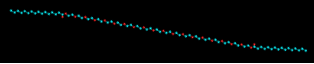  **(a)** |
| --- |
| 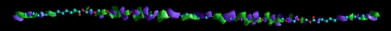  **(b)** |
| 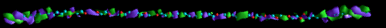  **(c)** |
| 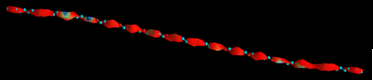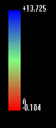  **(d)** |

Fig. S4: (a) SGC12 after optimization, (b) HOMO, (c) LUMO, and (d) Molecular electrostatic potential.

| 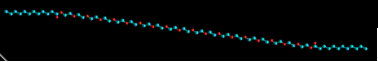  **(a)** |
| --- |
| 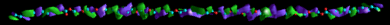  **(b)** |
| 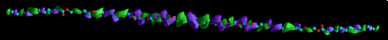  **(c)** |
| 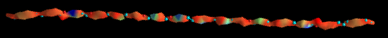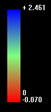  **(d)** |

Fig. S5: a) SGC16 after optimization, (b) HOMO, (c) LUMO, and (d) Molecular electrostatic potential.
